# Assessment of Hepatitis B Vaccination, Awareness, and Seroprotection Among Healthcare and Support Staff in a Tertiary Care Center in Central India

**DOI:** 10.7759/cureus.103395

**Published:** 2026-02-10

**Authors:** Rajeev K Jain, Deepti Chaurasia, Rakesh Shrivastava, Kamlesh K Ahirwar, Shailendra K Jain, Ankita Agarwal, Nagaraj Perumal

**Affiliations:** 1 Department of Microbiology, State Virology Laboratory, Gandhi Medical College, Bhopal, IND; 2 Department of Microbiology, Gandhi Medical College, Bhopal, IND; 3 Department of Gastroenterology, Gandhi Medical College, Bhopal, IND

**Keywords:** anti-hbs antibodies, healthcare workers, hepatitis b vaccination, india, occupational exposure, seroprotection, support staff, vaccination awareness

## Abstract

Introduction: Hepatitis B vaccination is a key strategy for preventing occupationally acquired hepatitis B virus infection among healthcare workers. However, variability in vaccination coverage, awareness, and post-vaccination serological monitoring persists across different professional groups in healthcare settings. This study assessed hepatitis B vaccination status, awareness, and seroprotection among healthcare and support staff in a tertiary care center in Central India.

Methods: A prospective cross-sectional observational study was conducted from January to June 2025 among healthcare workers and support staff at a tertiary care teaching hospital. Sociodemographic details, vaccination history, occupational exposure, and awareness were collected using a structured questionnaire. Serum anti-hepatitis B surface antibody levels were measured using an enzyme-linked immunosorbent assay. Seroprotection was defined as anti-HBs ≥ 10 mIU/mL. Associations were assessed using chi-square tests, and multivariate logistic regression was performed to identify predictors of seroprotection.

Results: A total of 676 participants were enrolled, including 429 (63.5%) healthcare workers and 247 (36.5%) support staff. Overall, 328 (48.5%) participants were fully vaccinated, 26 (3.8%) were partially vaccinated, and 322 (47.6%) were unvaccinated. The overall seroprotection rate was 359 (53.1%). Healthcare workers demonstrated higher vaccination coverage and seroprotection compared with support staff. Among fully vaccinated participants, 288 (87.8%) achieved seroprotection, while 40 (12.2%) were non-responders. Natural immunity was observed in a subset of unvaccinated participants. Vaccination status was the strongest independent predictor of seroprotection, and increasing age was associated with lower antibody response among fully vaccinated individuals. Self-reported vaccination awareness showed a significant association with verified seroprotection.

Conclusion: Hepatitis B vaccination coverage and seroprotection varied across professional groups, with comparatively lower levels among support staff. Strengthening inclusive occupational health strategies, improving vaccination documentation, and promoting post-vaccination antibody assessment may further enhance workforce protection in tertiary care settings.

## Introduction

Hepatitis B virus (HBV) infection remains a major global public health concern and continues to contribute substantially to occupationally acquired infections among healthcare workers (HCWs) [1]. Despite the availability of a safe and effective vaccine, healthcare personnel remain at risk due to frequent exposure to blood and body fluids during routine clinical and ancillary activities. This risk is particularly relevant in low- and middle-income countries, where adult immunization coverage and post-vaccination monitoring are often inconsistently implemented [2,3].

India is classified as an intermediate-endemic country for hepatitis B, with reported prevalence varying across regions and populations [4,5]. HCWs and hospital support staff, including ward attendants, housekeeping personnel, and security staff, are exposed to similar occupational hazards but often differ in access to structured vaccination programs and awareness initiatives. While hepatitis B vaccination is included under the Universal Immunization Programme for infants, vaccination coverage among adults in healthcare settings depends largely on institutional policies and occupational health practices and remains variably documented [6,7].

To address viral hepatitis as a public health priority, the Ministry of Health and Family Welfare, Government of India, launched the National Viral Hepatitis Control Program, which recommends hepatitis B vaccination for HCWs and other high-risk occupational groups [8]. The program also emphasizes the importance of maintaining vaccination records and conducting post-vaccination antibody testing to ensure adequate seroprotection. However, implementation of these recommendations varies across institutions, and routine serological monitoring is not uniformly practiced [9,10].

Protective immunity following hepatitis B vaccination is defined by an adequate anti-hepatitis B surface antibody response after completion of the recommended vaccination schedule. A proportion of vaccinated individuals may fail to develop or sustain protective antibody levels due to factors such as increasing age, comorbid conditions, improper vaccination schedules, or waning immunity [2,10]. Assessing seroprotection in addition to vaccination status is therefore important for understanding true occupational risk in healthcare settings.

Although several studies from India have examined hepatitis B vaccination coverage or awareness among healthcare workers [6,9,11], comprehensive institutional assessments that simultaneously evaluate vaccination status, awareness, and seroprotection across both clinical and non-clinical staff remain limited. Consequently, this study was undertaken with the following primary objectives: (i) to assess the current hepatitis B vaccination coverage among HCWs and support staff, (ii) to evaluate the level of awareness regarding hepatitis B transmission and prevention using a structured knowledge tool, (iii) to determine the seroprotection status (anti-HBs titers) of the staff and identify non-responders, and (iv) to characterize the virological profile (total anti-HBc and viral load) of seroprotected support staff to distinguish between vaccine-induced and natural immunity.

## Materials and methods

Study design and setting

This was a prospective cross-sectional observational study conducted over a six-month period from January to June 2025 at a tertiary care teaching hospital and its affiliated government healthcare facilities in Central India. The institution functions as a major referral center with diverse clinical services and a heterogeneous workforce, providing an appropriate setting to assess hepatitis B vaccination status, awareness, and seroprotection among healthcare and support staff.

Study population and sampling

The study population included full-time and outsourced contractual personnel employed at the institution for a minimum duration of six months. Eligible participants comprised clinical staff (consultants, resident doctors, nursing staff, laboratory and technical personnel, and medical or paramedical students) and non-clinical support staff (ward attendants, housekeeping personnel, sanitation workers, security personnel, and administrative staff). Written informed consent was obtained from all participants prior to enrollment.

Exclusion criteria included self-reported history of chronic liver disease, previously diagnosed HBV infection, current use of immunosuppressive therapy, and incomplete study data. Participants were recruited using a non-probability convenience sampling approach, wherein all eligible personnel present during the study period were invited to participate. This approach was chosen to maximize recruitment across diverse occupational categories within the resource-limited institutional setting.

Data collection and study variables

Data were collected through face-to-face interviews conducted by trained field workers using a pre-tested, structured questionnaire. To ensure inter-observer standardization, all field workers underwent a briefing session to standardize the interview process and reduce variability in data recording. The questionnaire was developed by the study authors based on standard guidelines. Content validity was established through review by a panel of subject matter experts in microbiology and gastroenterology. Furthermore, the tool was pre-tested on a pilot cohort (n = 10) to ensure the clarity and comprehensibility of questions before full implementation. It was administered in both English and Hindi. The English version of the questionnaire is provided in Appendix A.

The questionnaire captured information on sociodemographic characteristics, professional category, duration of service, hepatitis B vaccination history (number of doses received and documentation status), occupational exposure history, and awareness related to hepatitis B transmission, prevention, and vaccination. The awareness score was specifically derived from five items (Questions 15-19) covering transmission and prevention protocols.

Sample collection and laboratory testing

Venous blood samples (5 mL) were collected under aseptic precautions from all consenting participants using sterile, single-use vacutainers. Samples were transported to the State Virology Laboratory, Gandhi Medical College, Bhopal, India, where serum was separated by centrifugation and stored at -20°C until analysis.

**Figure 1:**
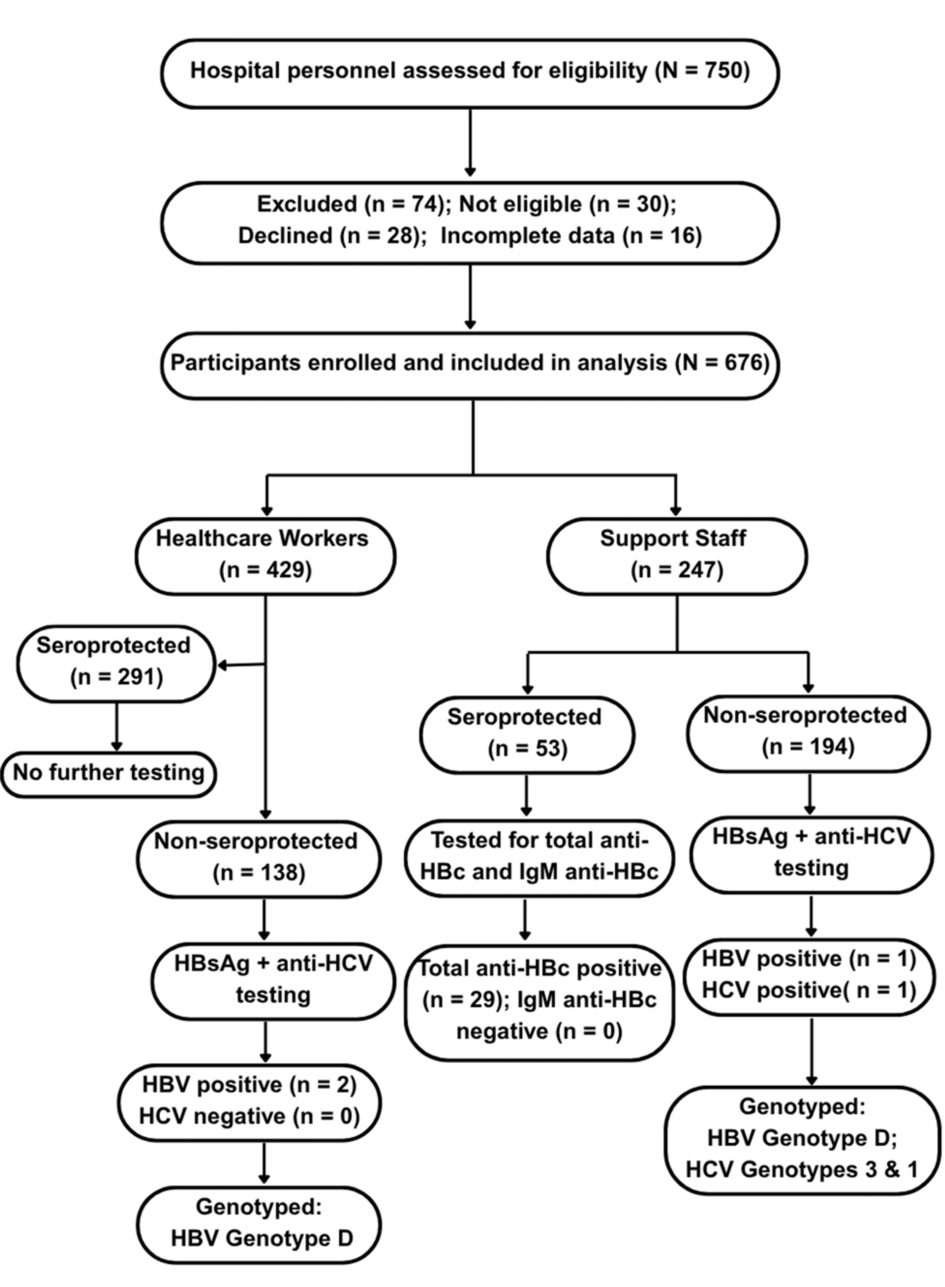
Participant enrollment, occupational classification, and virological testing flow diagram Flow diagram illustrating participant recruitment, eligibility screening, occupational classification, and virological testing algorithm. Of 750 hospital personnel assessed for eligibility, 676 were enrolled and included in the analysis. Participants were classified as healthcare workers (n = 429) or support staff (n = 247). All participants underwent screening for anti-hepatitis B surface antibodies. Non-seroprotected individuals were further tested for hepatitis B surface antigen and anti-hepatitis C virus antibodies. Among seroprotected support staff, additional testing for total anti-hepatitis B core antibody and IgM anti-hepatitis B core antibody was performed. Genotyping was conducted for hepatitis B virus and hepatitis C virus (HCV)-positive samples.

The sequential testing algorithm is illustrated in Figure 1. All serum samples were initially screened for anti-hepatitis B surface antibodies (anti-HBs) using a commercially available enzyme-linked immunosorbent assay (HBsAb ELISA kit; Dia.Pro Diagnostics Bioprobes Srl, Milano, Italy). An anti-HBs titer of ≥10 mIU/mL was considered indicative of seroprotection.

Participants with anti-HBs titers < 10 mIU/mL were further screened for hepatitis B surface antigen (HBsAg) using the Erba-Lisa PICO HBsAg ELISA kit (Transasia Bio-Medicals Ltd., India) and for anti-HCV antibodies using the HCV Microwell ELISA kit (Avantor Performance Materials India Pvt. Ltd., India).

Samples testing positive for HBsAg or anti-HCV were subjected to quantitative viral load estimation using the Truenat® HBV/HCV chip-based real-time polymerase chain reaction assay (Molbio Diagnostics, Goa, India). HCV genotyping was performed using the TRUPCR® HCV Genotyping Kit (3B BlackBio Dx Limited, Bhopal, India). HBV genotyping was carried out using a nested polymerase chain reaction method with genotype-specific primers, as previously described by Naito et al. [12].

To further characterize the virological profile of a vulnerable subgroup comprising support staff and ward attendants, participants who demonstrated seroprotection were additionally tested for total hepatitis B core antibody (anti-HBc) and IgM anti-HBc using Dia.Pro ELISA kits (Diagnostics Bioprobes Srl, Milano, Italy). All assays were performed strictly according to manufacturers’ instructions and under institutional quality control protocols.

Operational definitions

Participants were classified as fully vaccinated if they had received three doses of hepatitis B vaccine according to the recommended schedule (0, 1, and 6 months), partially vaccinated if they had received one or two doses, and unvaccinated if they reported no history or documentation of vaccination. Seroprotection was defined as an anti-HBs antibody titer of ≥10 mIU/mL. Non-responders were defined as fully vaccinated individuals with antibody titers below the protective threshold. Adequate awareness was defined as correctly answering at least 60% of the items in the awareness questionnaire.

Ethical considerations

The study protocol was reviewed and approved by the Institutional Ethics Committee of Gandhi Medical College, Bhopal, India (Approval No.: 43333/MC/IEC/2024). Written informed consent was obtained from all participants prior to enrollment. Participant confidentiality was maintained through anonymization of all collected data.

Statistical analysis

Data were entered into Microsoft Excel and analyzed using GraphPad Prism version 10.4.2 (Dotmatics, Boston, MA). Descriptive statistics were used to summarize participant characteristics, vaccination status, and serological findings. Associations between categorical variables were assessed using the Pearson Chi-square (χ²) test, with both χ² values and corresponding p-values reported.

Anti-hepatitis B surface antibody titers were log-transformed to calculate geometric mean titers (GMT). Binary logistic regression analysis was performed to identify independent predictors of seroprotection. Multicollinearity among predictors (e.g., professional role and education level) was assessed using variance inflation factors. A p-value of <0.05 was considered statistically significant.

## Results

Study participant recruitment and response rate

A total of 750 hospital personnel were initially screened for eligibility. Of these, 74 individuals were excluded: 30 did not meet the inclusion criteria, 28 declined to participate, and 16 provided incomplete data. This resulted in a final study cohort of 676 participants out of 720 eligible staff, yielding a high response rate of 93.9%. The recruitment flow, including exclusion details, is illustrated in the study participant flowchart (Figure 1).

Sociodemographic characteristics and overall status

Females constituted 426 (63.0%) participants, while 250 (37.0%) were male. The most common age group was 25-34 years, comprising 316 (46.7%) participants. HCWs comprised 429 (63.5%) participants, and 247 (36.5%) were categorized as support staff (Table 1 and Figure 1). Overall, 328 (48.5%) participants were fully vaccinated against hepatitis B, 26 (3.8%) were partially vaccinated, and 322 (47.6%) were unvaccinated. The overall seroprotection rate, defined as an anti-hepatitis B surface antibody titer ≥ 10 mIU/mL, was 359 (53.1%).

**Table 1:** Demographic profile, hepatitis B vaccination status, and seroprotection among hospital personnel (N = 676) Vaccination status: Participants were classified as fully vaccinated (receipt of three doses of the hepatitis B vaccine), partially vaccinated (receipt of one or two doses), or unvaccinated (receipt of zero doses or unknown vaccination history). Seroprotection status: Seroprotection was defined based on anti-hepatitis B surface antibody (anti-HBs) titers, with titers ≥ 10 mIU/mL considered seroprotective, and titers < 10 mIU/mL considered non-seroprotective. Statistical analysis: Associations between categorical variables were assessed using the Pearson Chi-square (χ²) test. The χ² statistic (with corresponding degrees of freedom) is reported in a separate column immediately preceding the p-value. A p-value < 0.05 was considered statistically significant and is indicated by an asterisk (*). For the professional category variable, a single omnibus χ² test was performed by simultaneously comparing all individual professional designations. Aggregated rows for healthcare workers and support staff are descriptive only and were not included separately in the χ² test calculations. Professional classification: Healthcare workers (N = 429) were defined as personnel with direct or frequent patient contact, whereas support staff (N = 247) included personnel with minimal or no direct patient contact.

Category	Subcategory	N (%)	Fully vaccinated, n (%)	Partially vaccinated, n (%)	Unvaccinated, n (%)	χ² value	p-value	Seroprotected, n (%)	Not Seroprotected, n (%)	χ² value	p-value
Gender	Female	426 (63.0)	204 (47.9)	17 (4.0)	205 (48.1)	0.21	0.898	224 (52.6)	202 (47.4)	0.16	0.686
	Male	250 (37.0)	124 (49.6)	9 (3.6)	117 (46.8)			135 (54.0)	115 (46.0)		
Age group (years)	<25	76 (11.2)	19 (25.0)	1 (1.3)	56 (73.7)	67.4	<0.001*	29 (38.2)	47 (61.8)	18.9	<0.001*
	25–34	316 (46.7)	175 (55.4)	17 (5.4)	124 (39.2)			177 (56.0)	139 (44.0)		
	35–44	189 (28.0)	81 (42.9)	7 (3.7)	101 (53.4)			101 (53.4)	88 (46.6)		
	≥45	95 (14.1)	53 (55.8)	1 (1.1)	41 (43.2)			52 (54.7)	43 (45.3)		
(A) Healthcare workers	Consultant	72 (10.7)	66 (91.7)	1 (1.4)	5 (6.9)	214.6	<0.001*	56 (77.8)	16 (22.2)	96.3	<0.001*
	Resident doctor	37 (5.5)	35 (94.6)	1 (2.7)	1 (2.7)			29 (78.4)	8 (21.6)		
	Nursing staff	99 (14.6)	82 (82.8)	14 (14.1)	3 (3.0)			67 (67.7)	32 (32.3)		
	Student	71 (10.5)	61 (85.9)	0 (0.0)	10 (14.1)			56 (78.9)	15 (21.1)		
	Technical staff	79 (11.7)	49 (62.0)	7 (8.9)	23 (29.1)			56 (70.9)	23 (29.1)		
	Ward boy	71 (10.5)	14 (19.7)	2 (2.8)	55 (77.5)			27 (38.0)	44 (62.0)		
	Subtotal	429 (63.5)	307 (71.6)	25 (5.8)	97 (22.6)			291 (67.8)	138 (32.2)		
(B) Support staff	Administrative	29 (4.3)	8 (27.6)	0 (0.0)	21 (72.4)			13 (44.8)	16 (55.2)		
	Housekeeping	108 (16.0)	10 (9.3)	1 (0.9)	99 (91.6)			25 (23.1)	83 (76.9)		
	Security	110 (16.3)	3 (2.7)	0 (0.0)	105 (95.4)			15 (13.6)	95 (86.4)		
	Subtotal	247 (36.5)	21 (8.5)	3 (1.2)	225 (91.0)			53 (21.5)	194 (78.5)		
Total	–	676 (100.0)	328 (48.5)	26 (3.8)	322 (47.6)	–	–	359 (53.1)	317 (46.9)	–	–

Vaccination status and seroprotection across professional categories

**Figure 2:**
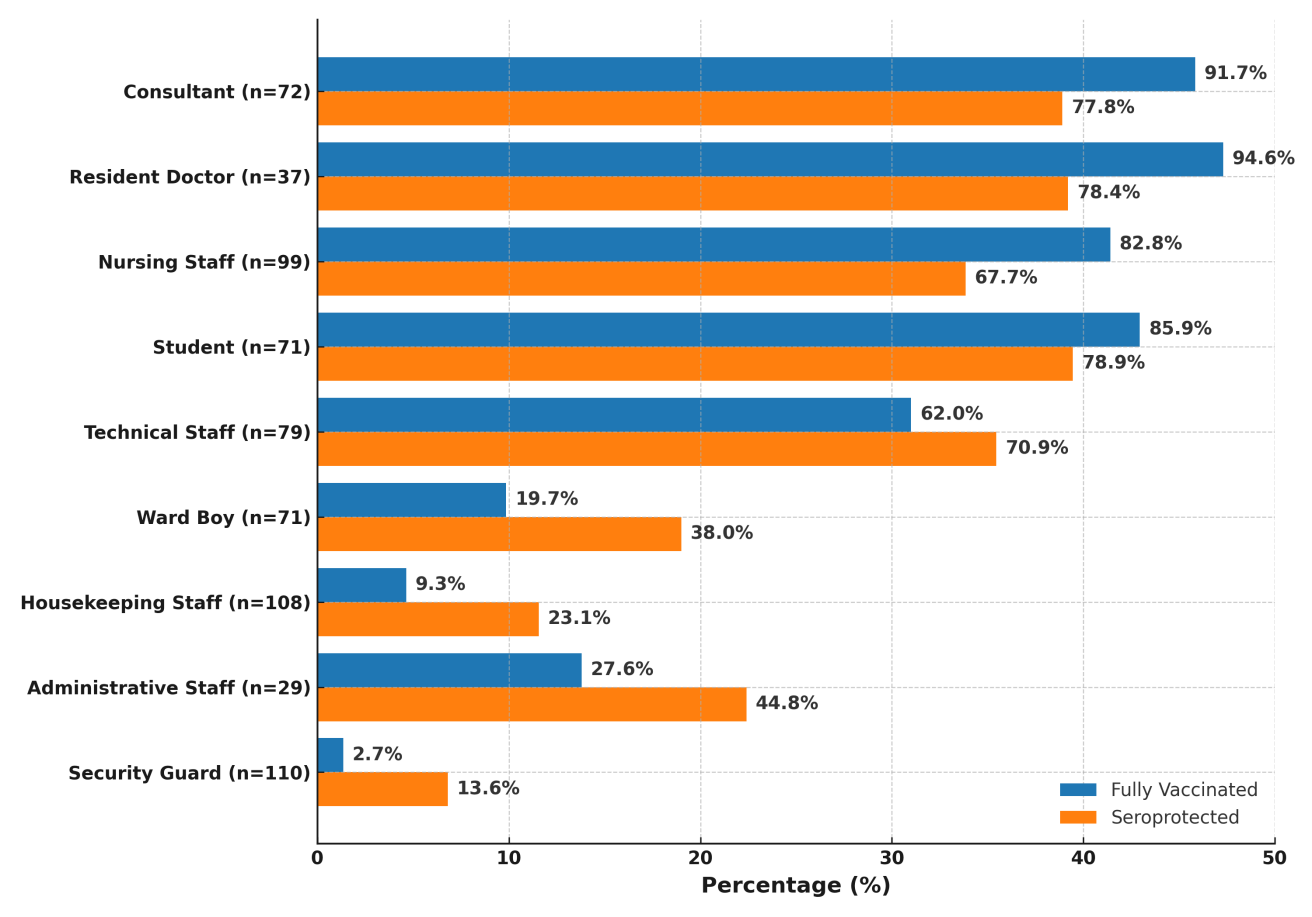
Hepatitis B vaccination and seroprotection status across professional categories Horizontal grouped bar chart depicts the proportion of personnel who were fully vaccinated against hepatitis B (blue bars) and those who demonstrated seroprotection based on anti-hepatitis B surface antibody titers ≥ 10 mIU/mL (orange bars), stratified by professional category. Percentages are shown for each subgroup, including clinical staff and non-clinical support staff.

Vaccination status and seroprotection varied significantly across professional categories and age groups (p < 0.001 for both) but not by gender (p = 0.898 and p = 0.686, respectively). Among HCWs, 307 (71.6%) participants were fully vaccinated, and 291 (67.8%) were seroprotected. In contrast, among support staff, 21 (8.5%) participants were fully vaccinated, and 53 (21.5%) were seroprotected (Figure 2). Full vaccination coverage was highest among resident doctors and consultants, while the lowest coverage was observed among security guards and housekeeping staff. Correspondingly, seroprotection was highest among students and lowest among security guards (Table 1 and Figure 2).

Distribution of anti-hepatitis B surface antibody titers

**Figure 3:**
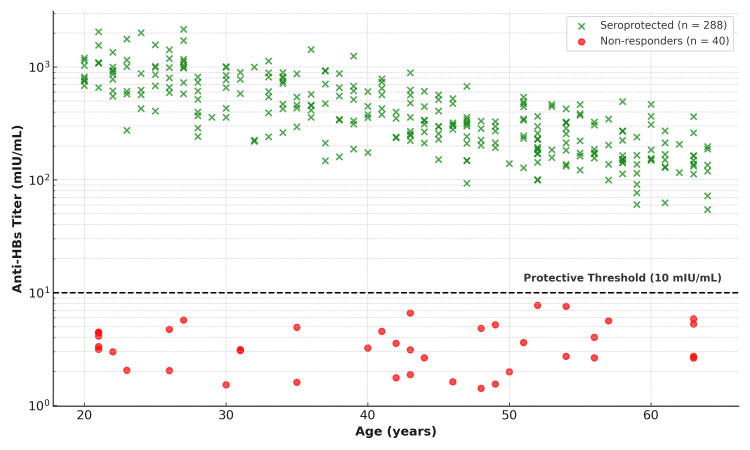
Anti-hepatitis B surface antibody titers by age among fully vaccinated participants This scatter plot illustrates anti-hepatitis B surface antibody titers (mIU/mL, logarithmic scale) plotted against age (years) among 328 fully vaccinated participants. Green cross-markers represent seroprotected individuals with antibody titers ≥ 10 mIU/mL (n = 288), while red circular markers represent non-responders with titers < 10 mIU/mL (n = 40). The horizontal dashed line denotes the seroprotective threshold of 10 mIU/mL.

The distribution of anti-hepatitis B surface antibody titers differed significantly by vaccination status (χ² = 338.9, df = 4, p < 0.0001) (Table 2). Among fully vaccinated participants (n = 328), 288 (87.8%) demonstrated seroprotective antibody titers (≥10 mIU/mL). High-level antibody titers (>100 mIU/mL) were observed in 225 (68.6%), while 63 (19.2%) had titers between 10 and 100 mIU/mL. Forty (12.2%) participants had antibody titers < 10 mIU/mL despite completion of the vaccination schedule; these individuals were categorized as non-responders, though this may also reflect waning immunity in those vaccinated in the distant past. Among partially vaccinated participants (n = 26), 11 (42.3%) demonstrated seroprotection. Among unvaccinated participants (n = 322), 281 (87.3%) had non-protective antibody titers, while 41 (12.7%) had titers ≥ 10 mIU/mL. Of these 41 participants, 36 (87.8%) were positive for total anti-hepatitis B core antibodies (anti-HBc), suggestive of naturally acquired immunity (Table 2). Age-wise distribution of antibody titers among fully vaccinated participants is shown in Figure 3.

**Table 2:** Distribution of anti-hepatitis B surface antibody titers by hepatitis B vaccination status among hospital personnel (N = 676) Anti-HBs titer interpretation: Titers were classified as non-protective (<10 mIU/mL), low-to-moderate protection (10–100 mIU/mL), and high-level protection (>100 mIU/mL). Geometric mean titer (GMT ± SD): GMT values (in mIU/mL) were calculated to summarize skewed antibody titer distributions; standard deviations were derived from log-transformed values. Statistical analysis: Associations between hepatitis B vaccination status and anti-HBs antibody titer categories were assessed using the Pearson Chi-square (χ²) test. The χ² statistic with corresponding degrees of freedom is reported in the table alongside the p-value. A p-value < 0.05 was considered statistically significant (*).

Vaccination status	n (%)	Anti-HBs antibody titers			GMT ± SD (mIU/mL)	χ² (df)	p-value
		<10 mIU/mL n (%)	10–100 mIU/mL n (%)	>100 mIU/mL n (%)			
Fully vaccinated	328 (48.5)	40 (12.2)	63 (19.2)	225 (68.6)	172.4 ± 2.4	χ² = 338.9 (4)	<0.001*
Partially vaccinated	26 (3.8)	15 (57.7)	10 (38.5)	1 (3.8)	36.8 ± 2.4		
Unvaccinated	322 (47.6)	281 (87.3)	21 (6.5)	20 (6.2)	61.5 ± 5.2		
Total	676 (100.0)	336 (49.7)	94 (13.9)	246 (36.4)	–		

Virological profile of the vulnerable group

A total of 318 participants were included in the vulnerable group, comprising ward boys and non-clinical support staff. Within this group, 247 (77.7%) participants had anti-hepatitis B surface antibody titers < 10 mIU/mL (Table 3). Total anti-HBc positivity indicating prior exposure was detected in 36 (11.3%) participants, with the highest proportion observed among housekeeping staff, followed by administrative and security personnel. Notably, all 36 anti-HBc-positive individuals were negative for IgM anti-HBc, suggesting past rather than acute infection. Screening of the non-seroprotected individuals within this subgroup identified one (0.3%) participant positive for HBsAg and one (0.3%) positive for anti-HCV. These low-prevalence findings were incidental, as the study was primarily powered to assess seroprotection rather than active infection prevalence. These findings are summarized in Table 3 and Figure 1.

**Table 3:** Virological profile of the vulnerable group (support staff and ward boys, N = 318) The vulnerable group comprised ward boys and non-clinical support staff, including administrative staff, housekeeping personnel, and security personnel, based on occupational categorization used for subgroup analysis. Hepatitis B surface antigen positivity and anti-hepatitis C virus antibody positivity indicate the presence of hepatitis B virus or hepatitis C virus infection, respectively. One participant tested positive for hepatitis B surface antigen and one participant tested positive for hepatitis C virus antibodies in this group. Anti-hepatitis B surface antibody titers were categorized as non-protective (<10 mIU/mL), low-to-moderate protection (10–100 mIU/mL), and high-level protection (>100 mIU/mL). Total anti-hepatitis B core antibody positivity indicates prior exposure to hepatitis B virus. IgM anti-hepatitis B core antibody positivity indicates recent or acute hepatitis B virus infection. All participants in this group tested negative for IgM anti-hepatitis B core antibody.

Designation	N	HBsAg positive, n (%)	HCV positive, n (%)	Anti-HBs Antibody titers			HBcAb positive, n (%)	HBc IgM positive, n (%)
				<10 mIU/mL, n (%)	10–100 mIU/mL, n (%)	>100 mIU/mL, n (%)		
Ward boy	71	0 (0.0)	1 (1.4)	50 (70.4)	4 (5.6)	17 (23.9)	8 (11.3)	0 (0.0)
Administrative staff	29	0 (0.0)	0 (0.0)	16 (55.2)	3 (10.3)	10 (34.5)	3 (10.3)	0 (0.0)
Housekeeping staff	108	0 (0.0)	0 (0.0)	86 (79.6)	7 (6.5)	15 (13.9)	17 (15.7)	0 (0.0)
Security guard	110	1 (0.9)	0 (0.0)	95 (86.4)	2 (1.8)	13 (11.8)	8 (7.3)	0 (0.0)
Total	318	1 (0.3)	1 (0.3)	247 (77.7)	16 (5.0)	55 (17.3)	36 (11.3)	0 (0.0)

Predictors of hepatitis B seroprotection

Multivariate logistic regression analysis identified vaccination status as the strongest independent predictor of seroprotection in the overall cohort (Table 4). Compared with fully vaccinated participants, partially vaccinated individuals had lower odds of seroprotection (adjusted odds ratio (AOR) = 0.081, 95% confidence interval (CI): 0.031-0.212, p < 0.001), while unvaccinated individuals demonstrated markedly reduced odds (AOR = 0.002, 95% CI: 0.000-0.013, p < 0.001).

**Table 4:** Multivariate logistic regression analysis of predictors of hepatitis B seroprotection Model A evaluated predictors of hepatitis B seroprotection, defined as anti-hepatitis B surface antibody titers ≥ 10 mIU/mL, in the full study cohort (N = 676). The reference categories for this model were fully vaccinated status and female gender. Model B evaluated factors associated with seroprotection failure, defined as anti-hepatitis B surface antibody titres <10 mIU/mL, among fully vaccinated participants (n = 328). The reference categories for this model were healthcare worker professional category and female gender. Adjusted odds ratios are presented with corresponding 95% confidence intervals. An adjusted odds ratio greater than one indicates increased odds of the specified outcome, while an adjusted odds ratio less than one indicates reduced odds. A p-value of <0.05 was considered statistically significant and is indicated by an asterisk.

Predictor variable	Adjusted odds ratio (AOR)	95% confidence interval (CI)	p-value
(A) Predictors of seroprotection in the overall population (N = 676)			
Vaccination status *(Ref: Fully Vaccinated)*			
Partially vaccinated	0.081	0.031–0.212	<0.001 *
Unvaccinated	0.002	0.000–0.013	<0.001 *
Age (per year increase)	1.012	0.993–1.031	0.214
Gender (Male vs. Female)	1.163	0.812–1.666	0.408
(B) Predictors of seroprotection failure among fully vaccinated personnel (n = 328)			
Age (per year increase)	1.054	1.016–1.093	0.005 *
Gender (Male vs. Female)	1.121	0.536–2.344	0.760
Professional role (Support Staff vs. HCW)	0.879	0.278–2.780	0.830

Among fully vaccinated participants (n = 328), increasing age was independently associated with seroprotection failure (AOR = 1.054 per year, 95% CI: 1.016-1.093, p = 0.005). While descriptive data showed disparities across job roles, professional category was not significantly associated with seroprotection failure in this multivariate subgroup analysis. This suggests that the observed differences between HCWs and support staff are largely mediated by their underlying vaccination coverage and awareness levels rather than their professional designation alone. Gender also showed no significant association with seroprotection failure (Table 4).

Association between self-reported vaccination awareness and seroprotection

Self-reported awareness of hepatitis B vaccination status was significantly associated with verified seroprotection status (χ² = 111.9, df = 2, p < 0.0001) (Table 5). Among participants who accurately reported being vaccinated (n = 192), 146 (76.0%) were seroprotected. Conversely, among those who reported being unvaccinated (n = 157), 122 (77.7%) were confirmed to be non-seroprotected. Notably, a large proportion of the cohort (n = 327, 48.4%) reported being unaware of their own vaccination status. Within this "unaware" group, 178 (54.4%) demonstrated seroprotection (Table 5).

**Table 5:** Association between self-reported vaccination awareness and verified seroprotection status (N = 676) Self-reported awareness: Based on participants’ responses to the question “Have you been vaccinated for hepatitis B?,” recorded as Yes, No, or Unknown. Seroprotection status: Defined as an anti-hepatitis B surface antibody (anti-HBs) titer ≥ 10 mIU/mL; titers < 10 mIU/mL were considered non-protective. Statistical analysis: The association between self-reported vaccination awareness and verified seroprotection status was assessed using the Pearson Chi-square (χ²) test. The χ² statistic with corresponding degrees of freedom is reported in the table alongside the p-value. The values shown represent a single omnibus χ² test comparing all awareness categories simultaneously. A p-value < 0.05 was considered statistically significant (*).

Self-reported awareness	n (%)	Seroprotected, n (%)	Not seroprotected, n (%)	χ² (df)	p-value
Yes (aware of being vaccinated)	192 (28.4)	146 (76.0)	46 (24.0)	111.9 (2)	<0.001*
No (believes unvaccinated)	157 (23.2)	35 (22.3)	122 (77.7)		
Unknown (unaware of status)	327 (48.4)	178 (54.4)	149 (45.6)		
Total	676 (100.0)	359 (53.1)	317 (46.9)		

## Discussion

This cross-sectional study provides a comprehensive institutional assessment of hepatitis B vaccination status, awareness, and seroprotection among healthcare and support staff in a tertiary care teaching hospital in Central India. Despite the availability of an effective vaccine and national recommendations advocating hepatitis B immunization for healthcare personnel, gaps were observed in vaccination coverage and protective immunity, particularly among non-clinical support staff.

In the present study, fewer than half of the participants were fully vaccinated. While HCWs showed higher coverage, substantial heterogeneity was observed across professional categories. Support staff, including housekeeping and security personnel, demonstrated the lowest coverage (8.5%). This pattern is consistent with other Indian studies [6,7,9] and highlights a critical gap in institutional occupational health; these individuals face similar risks from needle-stick injuries and environmental contamination but lack the protective benefits of structured vaccination programs.

A notable finding was that 12.2% of fully vaccinated individuals failed to demonstrate seroprotective antibody titers. Age emerged as a significant independent predictor of this failure. While increasing age is associated with an inherent decline in immune response, it is important to note that in our cohort, low titers in older participants may also reflect natural antibody waning over time rather than a primary failure to respond to the vaccine [2,10,13]. Similar observations have been reported in Indian tertiary care settings, where baseline and post-vaccination anti-HBs antibody titers showed considerable variability despite documented vaccination [14]. International data from Kuwait have also demonstrated that a proportion of vaccinated HCWs fail to maintain protective anti-hepatitis B surface antibody levels, underscoring the relevance of serological monitoring across different healthcare systems [15].

Among unvaccinated participants, 12.7% demonstrated seroprotective titers, most of whom were positive for total anti-HBc, suggesting naturally acquired immunity. In the support staff subgroup, the 11.3% anti-HBc positivity rate suggests a higher background exposure, likely due to lower awareness of standard precautions. Due to resource constraints, anti-HBc testing was prioritized for this vulnerable group to differentiate between vaccine-induced and natural immunity, though we acknowledge that the lack of data for clinical HCWs prevents a full cohort comparison. This finding is consistent with reports from intermediate-endemic regions, where background exposure to HBV has been documented among HBsAg-negative adults [4,5].

The virological assessment of the vulnerable subgroup revealed a low prevalence of active hepatitis B and hepatitis C infections. While these findings were incidental, as the study was primarily powered to assess seroprotection rather than infection prevalence, they highlight the potential presence of undiagnosed viral hepatitis among hospital personnel. This supports the implementation of targeted screening strategies within institutional occupational health programs to identify "silent" carriers among the staff.

Nearly half of the participants were uncertain about their vaccination status. Among these individuals, seroprotection was present in just over half, likely reflecting a combination of undocumented vaccination and natural immunity. This discrepancy underscores the limitations of relying solely on self-reporting for occupational health decision-making and justifies the need for objective serological monitoring [11].

The strengths of this study include the inclusion of both clinical and non-clinical staff, the use of laboratory-confirmed serological markers, and a robust sample size. However, several limitations must be acknowledged. The use of non-probability convenience sampling may limit generalizability to other settings, such as private or rural healthcare facilities. Vaccination history relied partly on self-report, introducing potential recall bias regarding the number and timing of doses.

Additionally, data on variables like BMI, smoking status, and specific comorbidities were not captured. Anti-HBc testing was restricted to the support staff subgroup due to resource constraints, which prevents a comprehensive comparison of naturally acquired immunity across the entire cohort. Finally, as a cross-sectional study, we cannot provide longitudinal data on the durability of seroprotection.

## Conclusions

This study demonstrates significant variability in hepatitis B vaccination coverage, awareness, and serological protection among hospital personnel in a tertiary care setting, with marked disparities observed among non-clinical support staff. Our findings highlight that documented vaccination status or self-report alone may not reliably reflect protective immunity, underscoring the necessity of incorporating objective serological assessment into occupational health protocols. Strengthening institutional strategies through the integration of centralized digital vaccination registries, periodic anti-HBs antibody monitoring, and targeted education programs is essential to bridge the immunity gap. Specifically, we recommend mandatory serological screening at the time of recruitment and the implementation of "catch-up" vaccination camps for support staff to align institutional safety with national hepatitis B control objectives.

## Appendices

Appendix A: Hepatitis B awareness and vaccination questionnaire (English version)

**Table 6:** Appendix A: Hepatitis B awareness and vaccination questionnaire (English version) This structured questionnaire was used to assess hepatitis B awareness, vaccination status, occupational exposure, and serological protection among hospital personnel. The questionnaire is an original tool developed by the authors for the present study. Credits: The authors of this study.

Study Title: Assessment of Hepatitis B Vaccination, Awareness, and Seroprotection Among Healthcare and Support Staff in a Tertiary Care Center in Central India			
Institution: Tertiary Care Teaching Hospital, Central India			
Ethics Approval: Approved by the Institutional Ethics Committee, Gandhi Medical College, Bhopal (IEC Protocol No.: 43333/MC/IEC/2024)			
Participant Code:			
Instructions: Participation is voluntary. All responses will be kept confidential and used only for research purposes. Please answer all questions honestly. If unsure, select “Not sure/Don’t know” where applicable			
Participant Information			
1	Age (in completed years):		
2	Gender:	☐ Male ☐ Female	
3	Department:		
4	Designation/Post:		
5	Job category:	☐ Healthcare worker (Consultant/Resident doctor/Nursing Staff/Medical/Paramedical Student/Technical Staff/Ward Boy)	
		☐ Support staff (Administrative Staff/Housekeeping Staff/Security Staff)	
6	Years of service in healthcare:	______ years	
Hepatitis B Vaccination History			
7	Have you ever received hepatitis B vaccination?	☐ Yes ☐ No ☐ Not sure	
8	If yes, how many doses have you received?	☐ One ☐ Two ☐ Three ☐ Not sure	
9	Do you have any written record of hepatitis B vaccination?	☐ Yes ☐ No	
10	Have you received a booster dose of hepatitis B vaccine?	☐ Yes ☐ No ☐ Not sure	
11	Year of last hepatitis B vaccine dose (if known):		
Occupational Exposure History			
12	Have you ever sustained a needle-stick or sharps injury during work?	☐ Yes ☐ No	
13	If yes, number of such injuries:		
14	Do you routinely use personal protective equipment (gloves, masks, and masks/face shields) while at work?	☐ Always ☐ Sometimes ☐ Never	
Awareness Related to Hepatitis B			
15	Hepatitis B can be transmitted through blood and body fluids.	☐ True ☐ False ☐ Do not know	
16	Hepatitis B vaccination can prevent infection.	☐ True ☐ False ☐ Do not know	
17	Healthcare and hospital support staff are at occupational risk of hepatitis B infection.	☐ True ☐ False ☐ Do not know	
18	A complete hepatitis B vaccination schedule consists of three doses.	☐ True ☐ False ☐ Do not know	
19	Protective immunity after hepatitis B vaccination can be confirmed by a blood test.	☐ True ☐ False ☐ Do not know	
Laboratory Assessment (Completed by Investigators)			
20	Anti-hepatitis B surface antibody titer (mIU/mL):		
21	Seroprotection status:	☐ Protective (≥10 mIU/mL) ☐ Non-protective (<10 mIU/mL)	
